# Characteristics of early and late onset pediatric depression

**DOI:** 10.1192/j.eurpsy.2023.1528

**Published:** 2023-07-19

**Authors:** L. Díaz-Castro, K. L. Hoffman

**Affiliations:** 1 Dirección de Investigaciones Epidemiológicas y Psicosociales, Instituto Nacional de Psiquiatría Ramón de la Fuente, Ciudad de México; 2 Centro de Investigaciones en Reproducción Animal (CIRA), Universidad Autónoma de Tlaxcala, Tlaxcala, Mexico

## Abstract

**Introduction:**

Depression, anxiety and suicide are serious psychiatric conditions that affect Mexican youth (Institute for Health Metrics and Evaluation, IHME, 2022), with depression showing a prevalence greater than 16%. Suicide ranks as the second most important cause of deathin this age group, (6/100,000 deaths), the first being violent deaths by firearms (15/100,000 deaths;IHME, 2022).

**Objectives:**

To identify factors related to age of onset of pediatric depression.

**Methods:**

A cross-sectional study was carried out during 2018-2020 in two Children’s Psychiatric Hospitals in Mexico City. Data were collected using a survey method. All participants signed an informed consent form. We applied Cox hazard analysis, with the hazard event being the onset of psychiatric symptoms.

**Results:**

Data from 400 patients were analyzed, 148 girls (37%) and 252 boys (63%). Mean patient age was 12 years, and mean age of symptom onset was 8 years. The most common diagnoses were hyperkinetic disorder (51%), depression (34%), and anxiety (7.8%). Age of depression onset was significantly reduced in association with male sex (HR=1.46), family history of psychiatric disorder (familial depression HR=2.34; hyperkinetic disorder HR=2.67; psychoactive substance abuse HR=5.09), and certain medical comorbidities (asthma HR=6.41; enuresis HR=3.03). These same covariates were not associated with age of onset of hyperkinetic disorder or anxiety.

**Conclusions:**

These analyses indicate that a subgroup of pediatric depression has an early onset and is associated with familial hyperkinetic disorder and depression, the male sex, and certain medical comorbidities.

**Disclosure of Interest:**

None Declared

